# Navigating bibliometric indicators in university rankings: a conceptual framework for strategic research management

**DOI:** 10.3389/frma.2026.1829439

**Published:** 2026-08-05

**Authors:** Wael Alkattan, Asma Terkawi, Mohamad Bakir, Mohammad Alaa Mufti, Karam Hamweyah

**Affiliations:** 1 Sustainability & Strategic Planning Office, Alfaisal University, Riyadh, Saudi Arabia; 2 Department of Medicine, College of Medicine, Alfaisal University, Riyadh, Saudi Arabia

**Keywords:** bibliometrics, citation analysis, field-weighted citation impact, normalized metrics, research evaluation, scopus, strategic planning, university rankings

## Abstract

The increasing prominence of global university rankings has fundamentally transformed the strategic landscape of higher education institutions worldwide. This review examines the strategic application of bibliometric indicators, with particular emphasis on Field-Weighted Citation Impact (FWCI), in the context of university ranking systems. We propose a framework for institutions to develop metric-informed strategies while maintaining research integrity and avoiding the pitfalls of metric manipulation. The review concludes with actionable recommendations for universities seeking to enhance their global standing through evidence-based research management practices.

## 1 Introduction

The state of global higher education has undergone a profound transformation over the past two decades, driven largely by the increasing influence of international university rankings (Hazelkorn, 2011). These rankings have transitioned from basic reputational surveys to sophisticated multi-dimensional evaluation systems that profoundly influence institutional strategy, financial allocation, and academic agendas (Marginson and van der Wende, 2007). Research performance metrics have become crucial indicators of institutional ranking, with citation-based measures exerting significant influence.

The Field-Weighted Citation Impact (FWCI) signifies a notable progression in bibliometric techniques, tackling enduring issues with the comparability of citation trends across various academic fields (Merton, 1979). In contrast to conventional citation counts or journal-based metrics, FWCI offers a normalized metric that facilitates equitable assessment of research impact across disciplines with significantly divergent publication and citation practices. This standardization is essential for institutional evaluation, as universities generally include many specialties with distinct citation practices.

Global universities are progressively acknowledging that their standings in international rankings significantly affect their capacity to attract premier talent, obtain research funding, forge international collaborations (MacRoberts and MacRoberts, 1989), and cultivate a robust research ecosystem. The quest for enhanced metrics must prioritize research integrity and prevent the inadvertent implications of metric optimization.

This paper seeks to present an overview of FWCI and associated bibliometric indicators within the framework of university ranking systems. We investigate the theoretical underpinnings and methodological aspects of these metrics, compare various bibliometric platforms and their citation normalization strategies, and evaluate how prominent ranking algorithms integrate these measurements. Additionally, we examine the strategic implications for institutions aiming to elevate their global prominence. This article adopts a narrative review and conceptual synthesis approach rather than a systematic literature review. By integrating bibliometric theory, institutional strategy, and critical perspectives on ranking systems, this paper aims to provide a framework for research administrators and academic leaders. The paper examines the interplay between citation-based indicators, institutional isomorphism, and the practical challenges of balancing global reputation with localized research missions.

## 2 Theoretical foundations of citation-based metrics

### 2.1 The evolution of citation analysis

Citation analysis, utilized for assessing research impact, originates from the foundational contributions of Eugene Garfield and the creation of the Science Citation Index in the 1960s (Schubert and Braun, 1986). The core principle of citation-based evaluation is that citations serve as a means of intellectual recognition, whereby researchers acknowledge and build upon the contributions of their colleagues. This framework regards citations as markers of academic impact and the dissemination of knowledge throughout the scholarly community.

The interpretation of citations as definitive indicators of quality or impact has been widely questioned. Merton's (1979) “Matthew Effect” in science explains how citation aggregation can be swayed by elements beyond study quality, such as author fame and institutional status (Merton, 1979). MacRoberts and MacRoberts (1989) delineated various limits in citation analysis, encompassing negative citations, ceremonial citations, and systematic biases in citation practices across disciplines (MacRoberts and MacRoberts, 1989). The creation of normalized citation metrics evolved in reaction to these criticisms, namely tackling the issue of cross-disciplinary comparison. The acknowledgment that citation rates significantly differ among disciplines, with biomedical sciences often exhibiting substantially greater citation frequencies than mathematics or humanities, prompted the creation of field-normalized measures (Schubert and Braun, 1986).

### 2.2 Field normalization: principles and approaches

Field normalization constitutes a significant methodological enhancement in bibliometric analysis, facilitating substantive comparisons across many study domains. The core premise entails evaluating the citation performance of a publication relative to a reference set of like publications, generally characterized by subject area, publication year, and document type (Waltman, 2016).

A variety of methodologies for field normalization have been established over the years. Source normalization evaluates citations in relation to the journal of publication, as demonstrated by the Source Normalized Impact per Paper (SNIP) measure (Moed, 2010). Field-based normalization evaluates against articles within the same subject category, serving as the foundation for measures like the Category Normalized Citation Impact (CNCI) and the Field-Weighted Citation Impact (FWCI) (Waltman et al., 2011a,b). Citation-based clustering employs citation networks to algorithmically delineate domains, rather than depending on established categories, hence providing a possibly more precise depiction of intellectual relationships (Boyack and Klavans, 2010). Every method possesses unique benefits and drawbacks. Journal-based normalization may provide challenges for multidisciplinary research or publications that encompass multiple disciplines. Field-based normalization is significantly reliant on the precision and detail of subject classification systems. Citation-based clustering, although theoretically more precise in delineating intellectual connections, can be computationally demanding and less visible in its approach (Leydesdorff and Bornmann, 2016).

### 2.3 Comparative methodology: FWCI, CNCI, and RCR

Field-Weighted Citation Impact (FWCI), Category Normalized Citation Impact (CNCI), and Relative Citation Ratio (RCR) are prominent metrics utilized in bibliometrics. All three serve as article-level field-normalized citation indicators, although they utilize distinct reference sets for normalization, resulting in varying biases. The primary sub-queries involve the normalizing technique, the resultant biases stemming from field delineation, and the tangible implications for assessment and comparison. FWCI and CNCI employ the traditional cited-side normalization method, wherein a paper's citations are split by the mean citations of comparable articles from the same year and discipline (Haunschild et al., 2022; Bornmann and Wohlrabe, 2019; Bornmann and Haunschild, 2017). In Scopus-based applications, the anticipated count is determined by document type, publication year, and subject area, usually categorized by ASJC, with papers allocated to various fields employing a harmonic mean over those fields. RCR employs a cited-side ratio, although its domain is dynamically established through the co-citation network of the manuscript instead of a predetermined journal category (Bornmann and Haunschild, 2017; Janssens et al., 2017). The numerator consists of citations annually, while the denominator represents an anticipated citation rate based on publications inside that co-citation-defined domain, benchmarked against NIH-funded peer-reviewed journals, resulting in a more focused and contextual comparison group than category-based metrics (Hutchins et al., 2016; Smith et al., 2023; Reddy et al., 2021).

FWCI and CNCI assess influence by comparing articles against averages derived from the same year of publication, document classification, and subject category (Huggett et al., 2018). Conversely, the RCR assesses annual citations relative to a dynamically established benchmark determined by the paper's unique co-citation network (Hutchins et al., 2016; Reddy et al., 2021). Moreover, in contrast to the universal category benchmarks employed by FWCI and CNCI, the RCR is contingent upon a standard uniquely associated with the performance of NIH-funded counterparts (Smith et al., 2023).

The primary methodological bias in FWCI and CNCI arises from the classification approach employed to designate fields. Conventional classification-oriented normalization is recognized to encounter issues, and outcomes can significantly vary when the same formula is utilized with diverse field-categorization frameworks (Haunschild et al., 2022; Waltman and Van Eck, 2013). This is not merely a theoretical concept: journals within the same substantive domain might be allocated disparate ASJC codes, resulting in papers on similar subjects being standardized against varying benchmarks (Scelles and Teixeira Da Silva, 2025). RCR was partially developed to mitigate this issue using co-citation-based field delineation, which is more effective at distinguishing fields than cited or citing networks and is more suitable for interdisciplinary research. However, RCR possesses its own predisposition: co-citation networks may encompass numerous loosely associated papers, and one criticism revealed that around 80% of co-cited articles were referenced together solely once, potentially rendering field delineation ambiguous and inconsistent (Janssens et al., 2017). Empirically, FWCI and RCR tend to have a strong correlation and demonstrate comparable stability across several disciplines, particularly at elevated aggregation levels like universities. Research on normalization techniques indicates that disparities among methods tend to diminish at larger scales, although they become increasingly significant for individual manuscripts, small collectives, journals, or niche disciplines (Waltman et al., 2011a,b; Zitt et al., 2005).

### 2.4 The sociology of citations

Comprehending citation behavior necessitates an examination of the social and cultural aspects that affect researchers' citation practices. Research has demonstrated systematic variations in citation habits throughout fields, career phases, and geographic regions (Borgman and Furner, 2002). Review articles generally generate more citations than original research publications, not solely due to superior research quality, but because they serve as thorough reference sources (Miranda and Garcia-Carpintero, 2018). The notion of “citation cultures” includes the standards and practices that regulate citation practice within particular academic communities. These cultures affect variables such as the average number of citations per publication, the inclination to reference contemporary vs historical literature, and the preference for self-citation or citation of proximate collaborators (Hellsten et al., 2007). Understanding these cultural disparities is crucial for accurately interpreting citation-based metrics and creating equitable grading systems that consider disciplinary variables outside the scope of mathematical normalization.

## 3 Field-weighted citation impact: methodology and implementation

### 3.1 FWCI calculation methodology

The Field-Weighted Citation Impact, utilized in Elsevier's Scopus database and SciVal analytics platform, is one of the most often employed field-normalized citation metrics. The computation employs a direct yet complex method in which FWCI is defined as the ratio of actual citations received to anticipated citations (Dresbeck, 2015). The actual citations denote the cumulative citations garnered by a publication within a designated timeframe, whereas expected citations indicate the mean citations accrued by analogous publications, characterized by identical publication year, document type, and subject area as delineated by the All Science Journal Classification (ASJC) system.

This methodology considers several elements that affect citation patterns. By adjusting for publication year, FWCI accounts for the inherent buildup of citations over time. Document type normalization acknowledges that reviews generally garner more citations than original research papers. Field normalization facilitates comparison among disciplines characterized by significantly disparate citation rates (Purkayastha et al., 2019).

### 3.2 Handling interdisciplinary research

A primary problem in field normalization relate to publications that encompass numerous disciplines. Scopus employs a fractional counting method when a publication is categorized under multiple ASJC classifications. The anticipated citations are computed as a weighted average derived from the citation trends within each pertinent discipline (Leydesdorff and Opthof, 2010). This methodology has significant ramifications for the assessment of transdisciplinary research. Publications situated in the convergence of high-citation and low-citation domains may leverage this averaging effect, thereby fostering cross-disciplinary collaboration. Nonetheless, genuinely innovative multidisciplinary research that does not conform to established classifications may be at a disadvantage if the reference sets fail to adequately reflect the citation trends of the developing subject (Rafols et al., 2012).

An institution's FWCI is primarily driven by research profiles that exhibit worldwide collaboration and multidisciplinary diversity, rather than dependence on a singular dominant discipline (Limaymanta et al., 2022; Li and Yin, 2023). Empirical studies indicate that articles categorized as “Multidisciplinary,” in addition to particular domains like Medicine and Physics, consistently achieve the highest FWCI scores among global universities (Limaymanta et al., 2022). This interdisciplinarity serves as a quantifiable catalyst for quality; for example, organizations exhibiting significant multidisciplinary variety (e.g., a Shannon index above 2.5) often attain overall FWCI values >1.5 (Li and Yin, 2023). Nevertheless, this approach necessitates meticulous adjustment, as an overabundance of topics within a single study group may ultimately diminish citation influence instead of augmenting it (Balel, 2025). Significantly, enhancing institutional FWCI is not just reliant on increasing total publishing output. Research demonstrates that concentrated scholarly quality often surpasses sheer size, enabling smaller institutions or departments to exceed their more prolific counterparts in normalized influence (Cheng et al., 2026). The development of these particular multidisciplinary and collaborative profiles acts as a significant catalyst for performance ranking; however, institutional strategists should be cognizant that FWCI may still be artificially skewed due to erratic or unreliable field classifications in bibliometric databases (Liu et al., 2021).

### 3.3 Temporal considerations and citation windows

The selection of the citation window profoundly influences FWCI values and their interpretation. Although Scopus computes the FWCI constantly, with updates occurring as new citations are added, the time of the evaluation is significantly important. Various disciplines display unique citation velocity patterns: biomedical and life sciences experience swift initial citation growth, peaking within 2–3 years; physical sciences and engineering show moderate citation velocity with consistent accumulation over 3–5 years; while mathematics and humanities reveal slow citation accumulation, typically necessitating 5–10 years for substantial evaluation (Wang, 2013).

These disparities require meticulous investigation when employing FWCI for institutional assessment. A one-year citation window may significantly underestimate influence in slow-evolving domains while potentially overstating early citations in rapidly advancing industries. Optimal methodologies include employing 3–5 year intervals for thorough evaluation, incorporating field-specific modifications as necessary (Abramo et al., 2011).

### 3.4 Statistical properties and reliability

The statistical characteristics of FWCI significantly influence its application in research assessment. The distribution of FWCI values is markedly skewed, with the majority of articles attaining FWCI values under 1.0 and a prolonged tail of extensively referenced studies. This skewness means that institutional FWCI averages can be significantly influenced by a small number of highly cited publications (Waltman and van Eck, 2019). The reliability of FWCI values also depends on the size of the reference set. For publications in narrow specialties with few comparable papers, the expected citation calculation may be based on small samples, leading to greater uncertainty. Institutions should be cautious when interpreting FWCI values for publications in emerging or niche fields (Thelwall and Wilson, 2014).

## 4 FWCI in university ranking systems

### 4.1 Times higher education world university rankings

The Times Higher Education (THE) World University Rankings exemplifies a significant utilization of FWCI in institutional evaluation. THE's methodology assigns 30% of the total score to research citations, rendering it a vital element of ranking performance. The implementation encompasses various intricate components (Zhou et al., 2024; Bautista-Puig et al., 2022).

The citation score calculation is based on a 5-year publication window using Scopus data with FWCI as the underlying metric, excludes publications with fewer than a threshold number of authors, and applies regional modification factors to account for citation biases. Previously, THE utilized granular indicators such as Citation Impact (15%), Research Strength (75th percentile of FWCI), Research Excellence (percentage of publications in top 10% by FWCI), and Research Influence (network-based) (Marginson, 2022).

The methodology has since evolved to consolidate these into a unified “Research Quality” pillar, mitigating issues associated with mean-based metrics distorted by outliers. Under this framework, the production of top 10% highly cited publications exerts a substantial influence on institutional performance. Specifically, THE directly evaluates “Research Excellence” by calculating the number of institutional publications landing in the global top 10 citation percentiles, normalized by publication year, subject category, and staff size (Kwan et al., 2024; Innocent, 2025). Consequently, universities that strategically cultivate these elite outputs not only maximize this targeted sub-indicator but also elevate their broader field-weighted citation metrics, which strongly correlate with upward ranking mobility (Szluka et al., 2023; Tóth et al., 2024). Benchmarking analyses confirm this pattern, revealing that top-ranked global institutions consistently couple a high overall FWCI with a disproportionately large share of top 10% outputs (Kwan et al., 2024). Nevertheless, relying exclusively on highly cited papers is a flawed institutional strategy. Because the THE methodology employs a multi-pillar evaluation framework, the ranking benefits of elite citation impact can be entirely offset by low overall publication volume, deficits in teaching or international outlook, or the influx of highly competitive new entrants (Innocent, 2025; Tóth et al., 2024). Therefore, while top 10% publications are a critical lever for maximizing citation-based scores, their impact is fully realized only when integrated into a comprehensive strategy that sustains robust research volume alongside performance across all methodological pillars (Rajagukguk et al., 2023).

### 4.2 Other ranking systems and citation metrics

Although THE notably incorporates FWCI, other significant ranking systems utilize alternative methodologies for citation analysis. The QS World University Rankings employs citations per faculty as its principal citation metric, derives data from Scopus without implementing field normalization, has faced criticism for potential bias against institutions in low-citation disciplines, and utilizes a more straightforward methodology that is less adept at addressing disciplinary variations (Hazelkorn and Mihut, 2021).

The Academic Ranking of World Universities (ARWU/Shanghai) incorporates highly cited researchers from the Clarivate Analytics list, utilizes publications in Nature and Science as indicators, applies various citation methodologies in discipline-specific rankings, and prioritizes absolute performance over normalized metrics (Liu and Cheng, 2005). The Leiden Ranking by CWTS is solely bibliometric, presenting several indicator alternatives, including both field-normalized and non-normalized metrics, enabling users to choose indicators and parameters, and exhibiting the highest transparency in process among prominent rankings (Waltman, 2016). The variety of methodologies underscores the absence of agreement on ideal citation metrics. Institutions must comprehend the technique of each system to formulate suitable strategies for enhancement (Daraio et al., 2016).

### 4.3 Regional and specialized rankings

In addition to worldwide rankings, other regional and specialty ranking systems utilize citation measures. The THE Asia University Rankings utilize a modified methodology that considers regional citation patterns, the QS Latin America Rankings apply adjusted weights to acknowledge regional publication habits, and U-Multirank, a European initiative, provides a multidimensional assessment (Hazelkorn and Gibson, 2017).

Subject-specific rankings frequently depend more on citation metrics because of disciplinary consistency, may utilize specialist databases, and can more effectively consider field-specific publication norms. These specialized rankings frequently offer a more detailed evaluation for universities with specific regional or disciplinary strengths. Nevertheless, they may be deficient in worldwide awareness and reputation compared to comprehensive world rankings (Aguillo et al., 2010).

### 4.4 Global rankings vs. local mission

A core conflict arises between enhancing global standings, which favor uniform results such as citation metrics, and addressing local societal objectives, which necessitate contextually relevant involvement (Mohamed Hashim et al., 2022; Drissi et al., 2025; Borodiyenko et al., 2023). As global frameworks urge universities to attain transnational prominence, regional advancement necessitates that institutions tackle particular topographical and socio-economic challenges (Thomas et al., 2023; Jones et al., 2021; Zenkiene and Leišyte, 2024; Fonseca and Nieth, 2021). This discrepancy is more pronounced when organizations encounter urgent societal demands. Arab colleges frequently emphasize urgent regional issues such as health, education, and unemployment (Saleh and Adly, 2024), whereas institutions in the Global South concentrate on localized sustainability (Drissi et al., 2025), crucial initiatives that are predominantly overlooked in international assessments.

Due to these conflicting demands, colleges are progressively embracing comprehensive roles in regional policy and community advancement (Thomas et al., 2023). Peripheral universities, especially, maintain significant interactions with local governments, and in times of crisis, this civic approach often takes precedence over research reputation as a key institutional focus (Fonseca and Nieth, 2021; Orzhel et al., 2023). Nonetheless, a continual operational discrepancy exists: although university strategic frameworks often advocate for regional accountability, their internal assessments and motivators seldom provide comprehensive backing for it (Borodiyenko et al., 2023).

Harmonizing these conflicting demands necessitates the reconfiguration of government and collaborations instead of viewing global standings and local involvement as opposing priorities (Zenkiene and Leišyte, 2024; Filho et al., 2023). Research suggests that collaboration across sectors, regional establishment, and deliberate involvement of stakeholders can simultaneously improve social effect and external recognition (Filho et al., 2023; Sribanasarn et al., 2025). In the end, colleges are evaluated based on international standards, yet their most significant impacts are frequently local, relational, and enduring, underscoring the persistent tension between quantifiable worldwide reputation and contextual societal worth (Veidemane, 2022; Hastangka Rahma et al., 2025).

## 5 Strategic applications and institutional implementation

### 5.1 Developing FWCI-informed institutional strategies

Universities aiming to improve their FWCI performance must formulate comprehensive strategies that match with their mission and capitalize on their existing strengths and areas of excellence. Effective execution necessitates synchronized efforts across several tiers. Universities should set explicit research performance objectives in accordance with strategic plans, invest in bibliometric infrastructure and expertise, implement incentive structures that prioritize quality over quantity, formulate policies that promote international collaboration, and allocate resources based on performance potential and strategic priorities.

International collaboration typically exhibits a robust positive correlation with normalized citation metrics like FWCI, while the extent of this advantage is significantly influenced by the strategic nature of the collaboration. Empirical investigations conducted in various international settings encompassing 60 institutions across five continents including public colleges in South America demonstrate that augmenting the number of nations included in a publication greatly enhances its FWCI (Limaymanta et al., 2022; Duarte et al., 2025). This increase is mostly fueled by articles co-authored abroad, which garner a significantly greater number of foreign citations, thus enhancing the research's visibility outside national limits (Wang et al., 2024). Nonetheless, optimizing FWCI cannot be accomplished with a simplistic “more is better” strategy concerning the number of countries. The caliber of partners is a vital factor; partnerships with economies that invest significantly in research are invariably linked to superior outcomes (Thelwall et al., 2024). Moreover, when cooperative networks consist of nations with very disparate research system capabilities, extensive international collaboration does not automatically surpass robust, concentrated regional studies (Vieira, 2023). Consequently, institutional approaches ought to transcend merely increasing the quantity of international collaborators, prioritizing the development of alliances that strategically enhance access to robust, high-investment research networks.

At the faculty and departmental level, implementation entails formulating a departmental research strategy that incorporates specific FWCI benchmarks, delivering consistent performance feedback via bibliometric dashboards, assisting early-career researchers in establishing high-impact research agendas, identifying research priorities, promoting interdisciplinary collaboration aligned with institutional strengths, and providing training on publication strategies and journal selection (Abramo et al., 2018).

Institutions should instruct individual researchers on citation metrics and their effects, offer a comprehensive guide to enhance awareness, provide tools and assistance for identifying high-impact publication venues, promote strategic collaboration with high-performing international partners, facilitate attendance at esteemed conferences for networking, and acknowledge and reward significant FWCI accomplishments in promotion considerations (Hicks et al., 2015).

### 5.2 Avoiding pitfalls and unintended consequences

The purposeful pursuit of enhanced metrics must be tempered by an understanding of possible adverse outcomes. Gaming and manipulation may occur through excessive self-citation or citation rings, fragmentation of research into numerous publications, evasion of dangerous or creative research that may not yield quick citations, and the disregard of teaching or service obligations in favor of publication pursuits (Biagioli and Lippman, 2020).

Excessive focus on FWCI may introduce disciplinary bias, disadvantaging professions with naturally lower citation rates or extended citation half-lives.

Field-normalized citation metrics, such the Field-Weighted Citation Impact (FWCI), may systematically penalize specialized research, applied sciences, and the humanities when baseline domains are inadequately defined, excessively broad, or derived from incomplete databases. In highly specialized niche domains, journals with overlapping substantive interests are often categorized into several subject classifications, leading to comparable articles being assessed against varying standards instead of a genuine peer group (Scelles and Teixeira Da Silva, 2025; Lisciandra, 2025). Moreover, expansive field classifications frequently do not effectively distinguish the unique citation practices of particular subfields (Lisciandra, 2025). The distinctive citation patterns of vertebrate paleontology are inaccurately portrayed when compared to wider ecological or geoscientific standards (Price, 2025). Applied sciences encounter further obstacles, as insufficient data and an absence of interconnected records in niche areas—like engineering or healthcare—make field-based normalization statistically precarious and untrustworthy (Szomszor and Adie, 2022).

The humanities and social sciences are especially disadvantaged by dependence on prominent bibliographic databases, which have traditionally shown inadequate representation of non-journal materials such as books and conference proceedings (Wilder and Walters, 2021; Worrall and Cohn, 2023). This disproportionate index coverage fundamentally distorts cross-disciplinary comparisons, as reliance on databases is a principal factor for citation metrics to vary significantly by discipline (Lisciandra, 2025). Moreover, research that is applied and socially engaged is systematically devalued as conventional metrics of publishing and citation do not adequately reflect a paper's wider impact on education, practical use, and public understanding (Wilder and Walters, 2021; Davies et al., 2021).

Notwithstanding these systemic drawbacks, it is crucial to acknowledge that the bias is not definitive; field-normalized citations continue to exhibit a favorable correlation with research quality across all disciplines, including the arts and humanities eight. Nonetheless, excessive dependence on these indicators may “over-simplify” specialty with varying average quality or citation practices, unintentionally masking achievement in transdisciplinary or ancillary domains instead of highlighting it (Thelwall and Jiang, 2025; Thelwall et al., 2023). As a result, measures such as FWCI often disadvantage research fields that are minor, diverse, inadequately indexed, or influential outside conventional journal formats, highlighting the importance of employing them with more comprehensive evaluation techniques instead of relying on them as isolated assessment instruments.

Institutions must ensure that evaluation systems account for disciplinary differences beyond what field normalization provides (Hammarfelt and de Rijcke, 2015). Ethical considerations are crucial, as the temptation to enhance metrics has been associated with a rise in scientific misconduct, including data fabrication and improper authoring procedures. Institutions must maintain strong research integrity frameworks alongside performance management systems (Fanelli, 2009).

## 6 Future directions and emerging trends

### 6.1 Technological advances in bibliometrics

Technological advancements are shaping the future of citation analysis and study appraisal. Artificial intelligence and machine learning facilitate the automated categorization of publications into precise subject classifications, forecasting future citation impact based on preliminary indicators, identifying citation manipulation and anomalous patterns, and employing natural language processing to comprehend citation context and sentiment (Fortunato et al., 2018).

The incorporation of artificial intelligence in research assessment presents considerable dangers of prejudiced and unclear evaluations that may compromise equity, diminish trust, and adversely influence researcher conduct (Thelwall, 2025). A major issue is the emergence of algorithmic, data, and user-interaction biases that might systematically punish certain demographics or academic disciplines instead of impartially evaluating quality. Despite seemingly strong technical performance, latent biases in big language models constitute a significant risk to fair assessment, a challenge intensified by unrepresentative training datasets that often overlook minority instances and academic contributions from the Global South (Pagano et al., 2023; Baumgartner et al., 2023). The absence of transparency exacerbates these bias issues. The opaque characteristics of numerous AI models hinder substantial independent evaluation; for example, an analysis of AI usage in oncology indicated that hardly 22% of research provided adequate data and repeatability resources, underscoring how lack of transparency impedes scientific validation (Smiley et al., 2025). Genuine transparency demands not only observable results but also thorough data lineage, ongoing oversight, and verifiable mechanisms. In the end, if AI instruments in bibliometrics are not meticulously scrutinized and consistently monitored by humans, they may provide inequitable rankings, enable unaccountable administrative choices, and foster new incentives for researchers to manipulate outcomes (Singhal et al., 2024).

Blockchain and distributed systems provide opportunities for transparent and immutable documentation of citations and metrics, decentralized methodologies for research assessment, and smart contracts for automated acknowledgment and reward mechanisms (Tenorio-Fornés et al., 2018). Real-time analytics are evolving from intermittent snapshots to continuous monitoring, incorporating dynamic dashboards with predictive analytics and early warning systems for performance deterioration (Rousseau et al., 2018).

### 6.2 Evolving evaluation frameworks

The research evaluation landscape is experiencing fundamental shifts in response to critiques of current systems. The responsible metrics movement, adhering to the San Francisco Declaration on Research Assessment (DORA) and the Leiden Manifesto, advocates for the utilization of quantitative metrics to complement, rather than replace, qualitative assessment. It acknowledges disciplinary variations in evaluation, recognizes a range of research outputs beyond journal articles, and emphasizes transparency in the computation and application of metrics (Hicks et al., 2015).

Multi-dimensional assessment methodologies will likely integrate various indicators, encompassing traditional citation metrics with enhanced normalization, altmetrics, societal impact measures, open science practices, reproducibility, collaboration and knowledge transfer metrics, as well as contributions to teaching and mentoring (European Commission, 2017). Instead of universal metrics, assessment may become more tailored to institutional missions, regional priorities, and disciplinary standards. This may result in more meaningful evaluations but complicate international comparisons (Ràfols, 2019).

### 6.3 Implications for global higher education

The advancement of research metrics and ranking systems significantly impacts the worldwide higher education environment. The growing stratification may occur as the focus on quantifiable research performance intensifies disparities between well-funded universities adept at strategic metric optimization and those lacking such resources. This may result in an increased concentration of resources and talent within already prestigious colleges (Cantwell and Taylor, 2013).

### 6.4 Institutional isomorphism and university rankings

Institutional isomorphism clarifies the ranking conduct of universities as an endeavor for legitimacy amongst shared external influences, compelling institutions to adopt like frameworks, agendas, and performance indicators (Stensaker et al., 2019; Fowles et al., 2016). In rankings, this typically entails modifying strategy, governance, and research endeavors to align with the indicators that denote worldwide standing, particularly citations, reputation, and international prominence (Szluka et al., 2023; Meylani, 2026; Peters, 2019).

Coercive isomorphism explains the process in which governments, accreditors, and policy frameworks associated with rankings compel universities to conform to approved organizational structures (Anafinova, 2020; Cardona Mejía et al., 2019). In the realm of higher education, this frequently entails the compulsion to embrace the Anglo-American research university paradigm, as prominent rankings favor research productivity and citations in prestigious English-language journals. Mimetic isomorphism elucidates the phenomenon whereby lower-status or uncertain colleges imitate the methods of more prestigious institutions in contexts where rankings delineate clear victors and vanquished. This emulation include the utilization of rankings in strategic development, the modeling of successful academic institutions, and adherence to recognized organizational frameworks for accreditation, programs, and research strategy (Anafinova, 2020; Cardona Mejía et al., 2019; Yoon et al., 2021). Normative isomorphism elucidates convergence via professional standards, prestigious faculty pathways, and global networks that propagate common perceptions of an exemplary university. In reality, colleges hire professors educated in prevailing research frameworks and use ranking criteria as assumed indicators of quality and credibility (Anafinova, 2020; Yoon et al., 2021; Adam, 2024).

**Figure 1:**
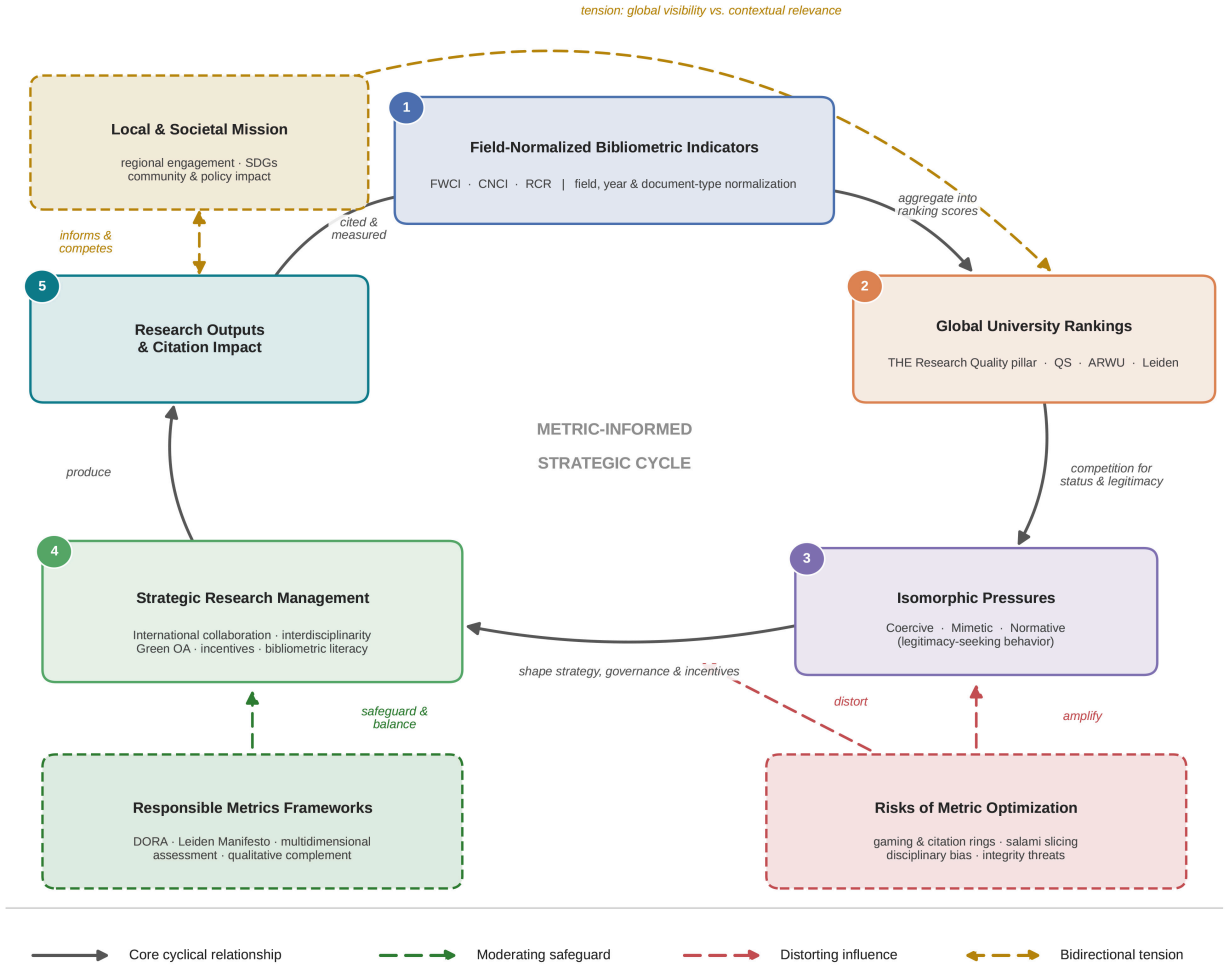
Conceptual framework of the metric-informed strategic cycle. Field-normalized bibliometric indicators (FWCI, CNCI, RCR) aggregate into global ranking scores, which generate coercive, mimetic, and normative isomorphic pressures that shape institutional research management strategies; the resulting research outputs feed back into the indicators. Responsible metrics frameworks (DORA, Leiden Manifesto) act as a safeguard on this cycle, while unchecked metric optimization introduces distortions (gaming, disciplinary bias, integrity risks). The institution's local and societal mission stands in bidirectional tension with globally oriented ranking performance.

Isomorphism does not yield absolute uniformity; responses are differentiated by status. Universities of varying ranks seek legitimacy through distinct approaches, although aligning with similar worldwide indicators (Stensaker et al., 2019; Adam, 2024). Rankings have significant stability and are contingent on prior paths, hence imitation may bolster established hierarchies instead of facilitating seamless transitions (Fowles et al., 2016; Peters, 2019; Sallam, 2025). This elucidates how ranking-driven isomorphism can constrict institutional diversity and skew academic conduct. Ranking systems streamline performance evaluation, encourage uniformity, and motivate focus on comparative standing, whereas publication-centric metrics might redirect research priorities toward enhancing visibility and maximizing citations (Maka et al., 2026). Recent findings additionally highlight more pronounced metric-responsive conduct at certain rapidly ascending colleges, encompassing authorship irregularities and heightened integrity-related threats (Meho, 2025) (Figure 1).

## 7 Recommendations for strategic implementation

Based on our comprehensive analysis, we propose the following recommendations for institutions seeking to strategically leverage FWCI and related metrics.

### 7.1 Strategic recommendations

By funding training programs for administrators, faculty, and researchers, setting up specialized bibliometric support units with technical know-how, and fostering an institution-wide awareness of metrics' advantages and disadvantages, institutions can cultivate comprehensive bibliometric literacy (Petersohn and Heinze, 2018). Balanced performance frameworks need to incorporate FWCI as a component within multi-dimensional assessment systems, tailor targets and benchmarks according to discipline and career stage, and prioritize research quality and integrity in conjunction with metrics (GläSer and Laudel, 2007).

Strategic alliances should be developed by discovering and nurturing partnerships with institutions, facilitating international mobility and exchange programs (Wagner and Leydesdorff, 2005). Investment in research infrastructure must ensure access to extensive bibliometric databases and tools, facilitate open access publishing in reputable settings, and establish institutional repositories to enhance exposure (Tennant et al., 2016).

Open Access (OA) publishing serves as a significant factor in the citation performance of institutions, while its influence on Field-Weighted Citation Impact (FWCI) is somewhat complex. Although open access journals typically achieve greater total citation counts and engage a more varied geographical and institutional readership, consistent FWCI improvements are not assured across every subject or open access format (Hadad et al., 2025). Empirical data suggests that Green OA, which employs repository-based access, exhibits the greatest significant citation benefit, consistently producing elevated FWCI scores and enhanced citation diversity compared to publisher-platform OA (Huang et al., 2024). Moreover, when journals consistently adopt open access models, they generally observe heightened publication rates and standardized citation influence, but these improvements differ significantly between disciplines (Momeni et al., 2021). It is essential that institutional open access plans are weighed against systemic equity risks. The shift to Open Access, especially those dependent on hybrid Article Processing Charges (APCs), may not ensure enhanced impact and may unintentionally exacerbate authorship inequality by limiting publishing opportunities for academics in economically disadvantaged areas (Chen et al., 2024; Levin et al., 2024). Thus, organizations aiming to strengthen FWCI ought to emphasize strong repository frameworks (Green OA) rather than universal mandates, guaranteeing that initiatives to improve global visibility do not undermine international collaborative fairness.

### 7.2 Policy and governance recommendations

Policymakers and ranking bodies should enhance methodological precision to improve classification methods for new disciplines, devise superior strategies for interdisciplinary research, and rectify acknowledged biases in citation databases and practices. Transparency must be enhanced by mandating comprehensive disclosure of ranking techniques and data sources, facilitating institutional verification of their data, and disseminating confidence intervals and indicators of ambiguity.

Responsible use should be encouraged through developing guidelines for appropriate metric application, discouraging over-reliance on single indicators, and supporting research into evaluation methodologies (de Rijcke et al., 2016). Specialized measures should be developed for various regional and disciplinary contexts, instead of uniform evaluation methods and acknowledging unique paradigms of research achievement.

## 8 Conclusion

The Field-Weighted Citation Impact has become an effective instrument for research evaluation, providing substantial benefits above conventional citation counts due to its advanced normalization methodology. The FWCI's significant position in esteemed rating systems such as Times Higher Education has become it an essential statistic for institutional strategy and research administration. Our analysis indicates that although FWCI mitigates certain shortcomings of previous citation metrics, it possesses its own limitations and potential for misuse. The strategic utilization of FWCI and associated metrics necessitates a sophisticated comprehension of their technical characteristics, recognition of their limitations, and dedication to responsible execution. Institutions must avoid the inclination to prioritize metric optimization over research quality, integrity, and overarching academic principles. The most effective strategies will utilize metrics as instruments to promote institutional excellence, rather than viewing them as objectives in their own right. The primary objective of any metric system should be to foster and acknowledge exemplary research that enhances knowledge and serves societal interests. By sustaining this viewpoint while selectively utilizing bibliometric indicators, institutions can increase their global reputation in ways that bolster rather than undermine their core academic goals. The forthcoming issue involves managing the conflict between competitive demands and academic principles, use metrics judiciously to cultivate research environments that are both outstanding and sustainable. The primary objective of any metric system should be to foster and acknowledge outstanding research that enhances knowledge and serves societal interests. By adopting this viewpoint and deliberately utilizing bibliometric indicators, institutions can increase their global reputation in ways that support rather than undermine their core academic objectives. The forthcoming challenge involves managing the conflict between competitive pressures and academic principles, use metrics judiciously to cultivate research environments that are both exemplary and sustainable.
